# Structural Determination of Three Different Series of Compounds as Hsp90 Inhibitors Using 3D-QSAR Modeling, Molecular Docking and Molecular Dynamics Methods

**DOI:** 10.3390/ijms12020946

**Published:** 2011-01-30

**Authors:** Jianling Liu, Fangfang Wang, Zhi Ma, Xia Wang, Yonghua Wang

**Affiliations:** 1 College of Life Sciences, Northwest University, Xi’an, Shaanxi 710069, China; E-Mails: ljl2003ljl@126.com (J.L.); yu100288@163.com (F.W.); 2 Center of Bioinformatics, Northwest A&F University, Yangling, Shaanxi 712100, China; E-Mails: mshappy@126.com (Z.M.); fishery18@163.com (X.W.)

**Keywords:** Hsp90, 3D-QSAR, CoMFA, CoMSIA, molecular docking, molecular dynamics

## Abstract

Hsp90 is involved in correcting, folding, maturation and activation of a diverse array of client proteins; it has also been implicated in the treatment of cancer in recent years. In this work, comparative molecular field analysis (CoMFA), comparative molecular similarity indices analysis (CoMSIA), molecular docking and molecular dynamics were performed on three different series of Hsp90 inhibitors to build 3D-QSAR models, which were based on the ligand-based or receptor-based methods. The optimum 3D-QSAR models exhibited reasonable statistical characteristics with averaging internal *q*^2^ > 0.60 and external *r*^2^_pred_ > 0.66 for Benzamide tetrahydro-4*H*-carbazol-4-one analogs (BT), AT13387 derivatives (AT) and Dihydroxylphenyl amides (DA). The results revealed that steric effects contributed the most to the BT model, whereas H-bonding was more important to AT, and electrostatic, hydrophobic, H-bond donor almost contributed equally to the DA model. The docking analysis showed that Asp93, Tyr139 and Thr184 in Hsp90 are important for the three series of inhibitors. Molecular dynamics simulation (MD) further indicated that the conformation derived from docking is basically consistent with the average structure extracted from MD simulation. These results not only lead to a better understanding of interactions between these inhibitors and Hsp90 receptor but also provide useful information for the design of new inhibitors with a specific activity.

## 1. Introduction

Hsp90 belongs to a family of proteins called molecular chaperones that are responsible for maintaining the appropriate folding and three-dimensional conformation of proteins in the cell and are critical for controlling the balance between the synthesis and degradation of many proteins. Furthermore, they have also been shown to play an important role not only in the stress response but also in regulating many critical cellular functions, such as cell proliferation and apoptosis [1–3]. More recently, Hsp90 has emerged as a very important and validated target in several diseases including cancer, neurodegeneration, viral, fungal, and microbial infection [4–14]. Therefore the development of Hsp90 inhibitors has become an urgent field in both theoretical and practical fields, in view of the urgency for improving cancer treatment in recent years.

Hsp90 exists as two isoforms: Hsp90α and Hsp90β [15]. In this study, we adopted Hsp90α as the target as Hsp90α is an inducible form overexpressed in cancer cells. Hsp90 consists of a highly conserved *N*-terminal domain, a charged linker, and a highly conserved *C*-terminal region, the charged linker is not important for Hsp90 function, and the inhibitors of Hsp90 mainly bind to the *N*-and *C*-terminal regions [16–18]. These inhibitors first act on Hsp90, then disrupt client proteins folding and degradation via the ubiquitin-proteasome pathway. Since Hsp90 client proteins are signaling proteins implicated in survival/proliferation, including protein kinases, antiapoptotic proteins and transcription factors, its inhibition has the potential to simultaneously interfere with multiple signaling pathways [16–18]; meanwhile, these proteins are important for tumor cell survival. The *N*-terminal domain binds the natural products geldanamycin (GDA), radicicol and their derivatives, which modulate at least two different conformational states. Novobiocin binds to the *C*-terminal nucleotide binding site and inhibits Hsp90 function. In this work, we mainly focus on uncovering the interaction mechanism of inhibitors in the *N*-terminal site of the protein.

The first Hsp90 inhibitor drug is geldanamycin, a natural product isolated from Streptomyces hygroscopicus [19]. Although geldanamycin displays high potency in cytotoxicity assays, it also shows hepatotoxicity in preclinical trials [20]. Interestingly, the geldanamycin analogue 17AAG (17-allylamino-17-demethoxy-geldanamycin) possesses all the Hsp90-related characteristics of geldanamycin but with lower toxicity [19,21,22]. Although less hepatotoxic than geldanamycin, 17-AAG is poorly soluble, difficult to formulate and undergoes extensive metabolism leading to non-target-based effects [21]. Another natural Hsp90 inhibitor is radicicol (RD), which has high binding affinity, but limited *in vivo* stability. Besides these natural compounds, some synthesized inhibitors have also shown promising effects on Hsp90, for example, purine-based inhibitors [23,24], the Pyrazole-isoxazole analogues, Novobiocin and coumarin scaffold analogues, such as 4TCNA [25]. The deficiency of natural compounds led to significant efforts to identify novel small molecule inhibitors of Hsp90 which had more potent inhibitory activity and could ideally be fitted for combination therapies for cancer. To date, a number of Hsp90 inhibitors have been reported [26–28]. More recently, the 3D-QSAR (3 dimensional-quantitative structure-activity relationship) including CoMFA, CoMSIA and 3D-pharmacophore and docking methods were employed to investigate PU3 analogues [29,30], which provided useful models for designing the Hsp90 targeted inhibitors. In addition, another work has described an integrated 3D-QSAR model using pharmacophore modeling and docking approaches applied on a dataset of 72 Hsp90 adenine inhibitors [31]. The results found a set of pharmacophoric features, *i.e.*, one H-bond donor, one H-bond acceptor, one hydrophobic_aromatic, and two hydrophobic_aliphatic features, which might be of import to develop inhibitors as anti-cancer agents [31]. At the same time, some other series of compounds, such as Benzamide tetrahydro-4*H*-carbazol-4-one analogs (BT) [32], AT13387 derivatives (AT) [33] and Dihydroxylphenyl amides (DA) [34], have been developed as further potent Hsp90 inhibitors. In these literatures, the authors only synthesized and evaluated the inhibitors; to date, there are no three-dimensional quantitative relationship (3D-QSAR) studies of the ligands reported. Moreover, no comprehensive feature for the ligand-receptor interactions, such as the hydrophobic contact between the key amino acid residues has been demonstrated. Therefore, in order to investigate the binding mechanism and the properties of these inhibitors, we have studied the quantitative structure—activity relationships of these three classes of Hsp90 inhibitors. In this work, we used the comparative molecular field analysis (CoMFA) [35] and comparative molecular similarity indices analysis (CoMSIA) methods [36] to produce 3D-QSAR models with the ligand-based and receptor-based approaches for three different classes of Hsp90 inhibitors. Ligand-based 3D-QSAR approaches were reported to be effective for understanding the structure—activity relationships [37]. While the more reliable receptor-based 3D-QSAR methods were also employed to demonstrate the physical-chemical principles of the interactions between inhibitors and Hsp90. Moreover, the contour map generated from the CoMFA and CoMSIA models can further illustrate the observed variation in activity for the given structural differences in these inhibitors. In addition, molecular dynamics was performed to better understand the probable binding modes of these inhibitors. The developed models can, not only be used to predict the activities of new designed inhibitors, but also to provide useful information for modification in the design of new potential inhibitors.

## 2. Material and Methods

### 2.1. Data Sets and Biological Activity

The 89 compounds involved in this study were taken from [32–34]. Those molecules that did not have the specified inhibitory activity and did not share a common scaffold with the other molecules were discarded. Inhibitory concentrations *K*_d_ (μM) and *IC*_50_ (μM) of the molecules were converted into corresponding inhibitory activity p*K*_d_ (−log*K*_d_) and p*IC*_50_ (−log*IC*_50_) values, which were used as dependent variables in the 3D-QSAR analysis. The total set for each group was divided into the training set and the test set. The test set was selected by considering that the test compounds represent structural diversity and a range of biological activities similar to that of the training set. In addition, the training set was used to generate 3D-QSAR models, while the test set was employed to validate the quality of the models. All the chemicals and activities are presented in Tables S1–S3.

All molecular modeling was performed using SYBYL package (Tripos Associates, St. Louis, MO, U.S.). 3D structures of all molecules were constructed using the Sketch Molecule function in Sybyl software. Gasteiger-Hückel charges were employed to calculate the partial atomic charges. Energy minimization was performed by using the Tripos force field [38] with the Powell conjugate gradient minimization algorithm and a convergence criterion of 0.05 kcal/mol·Å.

### 2.2. Conformational Sampling and Alignment

**Figure 1.**
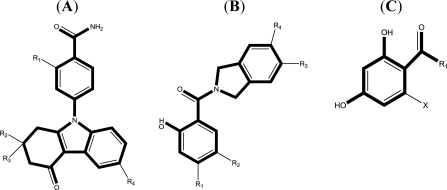
Skeletons for benzamide tetrahydro-4*H*-carbazol-4-one analogs (**A**); AT13387 Derivatives (**B**) and dihydroxylphenyl amides (**C**).

**Figure 2.**
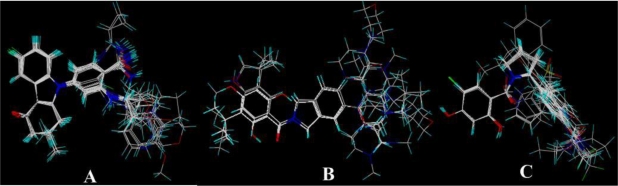
Ligand-based alignment. (**A**) Compound 17 was used as a template for alignment; (**B**) Compound 24 was used as a template for alignment; (**C**) Compound 19 was employed as a template for alignment. C is colored in white; H is colored in cyan; O is colored in red; N is colored in blue; S is colored in yellow.

**Figure 3.**
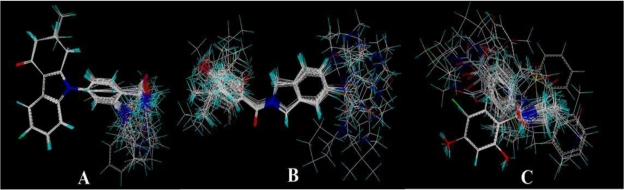
Receptor-based alignment. (**A**) Compound 17 was selected as a template for alignment; (**B**) Compound 24 was used as a template for alignment; (**C**) Compound 19 was employed as a template for alignment. Alignment of these compounds was further used for 3D-QSAR model generation.

In the 3D-QSAR studies, in order to obtain valid and reliable models, molecular alignment was employed. In this study, two alignment rules were employed; one is ligand-based alignment. In this method, a molecule of each class that has the most potent inhibitory activity (compounds 17, 24, 19, respectively) was chosen as template molecule to align with the remaining training and test compounds by using the Database Align function in SYBYL. The common substructure of the compounds is shown in bold in Figure 1 and the aligned compounds are depicted in Figure 2. The other approach is the receptor-based alignment, in which the aligned results are further applied to QSAR analysis. The receptor-based alignment is shown in Figure 3.

### 2.3. 3D-QSAR Analysis

CoMFA and CoMSIA studies were performed based on the molecular alignment as described above in order to build predictive 3D-QSAR models. CoMFA calculates steric and electrostatic properties, whereas CoMSIA calculates similarity indices in the space surrounding each of the molecules in the dataset.

The aligned molecules were placed in a 3D grid box of 2.0 Å such that the entire set was included in the simulation box. The CoMFA descriptors, steric field and electrostatic fields with a distance-dependent dielectric at each grid point were calculated using a sp3 carbon atom probe with a vander Waals radius of 1.52 Å and a charge of +1.0 using default settings in SYBYL. The CoMFA fields generated automatically were scaled by the CoMFA-STD method with default energy of 30 kcal/mol.

In addition to steric and electrostatic fields, hydrophobic, hydrogen-bond donor and acceptor descriptors were calculated in CoMSIA models. CoMSIA similarity index descriptors were derived using the same lattice box as that used in the CoMFA calculations. All the five physicochemical descriptors were calculated using the standard settings that probe with charge +1, radius 1 Å and hydrophobicity +1, hydrogen-bond donating +1, hydrogen-bond accepting +1, attenuation factor α of 0.3 and grid spacing 2 Å. CoMSIA similarity indices (*A_F_*) for a molecule *j* with atoms *i* at a grid point *q* are calculated by Equation (1) as follows:

(1) $$A_{\text{F},\text{K}}^{q}(j)=-{\sum \omega_{\text{probe},k}\omega_{ik}e_{iq^{2}}}^{-\alpha r}$$

Five physicochemical properties *k* (steric, electrostatic, hydrophobic, hydrogen bond donor, and hydrogen bond acceptor) were evaluated using the common sp^3^ carbon probe atom. *W_ik_* is the actual value of physicochemical property *k* of atom *i*, and *W*_probe,_*_k_* is the value of the probe atom. α is the attenuation factor and the default value of 0.3 was used. A Gaussian type distance dependence was used between the grid point *q* and each atom *i* of the molecule. This can avoid singularities at the atomic positions and the dramatic changes of potential energy due to grids in the proximity of the surface [39].

The CoMFA/CoMSIA fields combined with observed biological activities (p*IC*_50_) were included in a molecular spreadsheet and partial least square (PLS) methods [40] were applied to generate 3D-QSAR models. The cross-validation analysis was performed using the leave-one-out (LOO) method. The cross-validated *r*^2^ which was used to generate the optimum number of components and lowest standard error of prediction were taken. Then the optimum number of components obtained from the cross-validation analysis was used to calculate the conventional *r*^2^. The predictive ability of the 3D-QSAR model was determined using the compounds which were not included in the training set. The predictive correlation coefficient *r_pred_*^2^, based on the test set molecules, was calculated using Equation (2):

(2) $$r_{\text{pred}}^{2}=(\text{SD}-\text{PRESS})/\text{SD}$$

where, *SD* is the sum of the squared deviations between the biological activities of the test set and mean activities of the training set molecules and PRESS is the sum of squared deviation between predicted and actual activities of the test set compounds.

### 2.4. Molecular Docking

Molecular docking is an application wherein molecular modeling techniques are used to predict how a protein (enzyme) interacts with small molecules (ligands) [41]. Molecular docking was performed to study the binding modes for the allosteric site of Hsp90 protein with its ligands and to develop docking-based 3D-QSAR models. All the parameters were set as the default values in the whole process. The crystal structures of Hsp90 have been obtained from RCSB protein data bank [42] (3D0B, 2XJG and 3K97). During the procedure, two parameters, *i.e.*, protomol_bloat and protomol_threshold, which can determine how far from a potential ligand the site should extend and how deep into the protein, the atomic probes used to define the protomol can penetrate, are specified default values. Finally, each conformer of all 89 inhibitors in three different groups, was docked into the binding site 10 times. During the process, water molecules and the inhibitors were removed and hydrogens were added to the system. The putative poses of molecules were scored using the Hammerhead scoring function [43], which also served as an objective function for local optimization of poses. On the other hand, the ligands (*i.e.*, substrates) were regarded as being flexible, while the enzyme was regarded as rigid. Finally, the highest-ranking conformers/poses, according to each scoring function, were aligned together for CoMFA and CoMSIA modeling.

Before docking analysis, a redocking process was performed to verify that the docking parameters specified in the input file for docking method were reasonable, and able to recover a known complex’s structure and interactions. The root-mean-square deviation (RMSD) values are between 0.7 and 1.6 Å for the 10 top-ranked docking poses for BT, between 0.6 and 1.9 for AT and between 0.08 and 0.6 for DA.

### 2.5. Molecular Dynamics Simulations

All MD simulations were performed with the GROMACS program package [44], employing the GROMOS96 force field [45]. For MD simulations, the three models (BT, AT and DA) used NPT ensemble at 300 K with periodic boundary conditions, and the temperature was kept constant by a Berendsen thermostat; the values of the isothermal compressibility were set to 4.5 × 10^−5^ bar^−1^. Van der Waals interactions and cut-off distances for the calculation of Coulomb were 1.4 and 1.0 nm, respectively. The particle mesh Ewald method [46] was used to calculate electrostatic interactions.

For this simulation, each system applied a cubic periodic box with a side length of 70.55 Å, 66.81 Å and 74.77 Å, respectively. Between the protein and the box walls the minimum distance was set to more than 8 Å. The net charge of the protein was neutralized by 8 Na^+^, 11 Na^+^ and 8 Na^+^ for BT, AT, DA series of Hsp90 inhibitors. The extended simple point charge (SPCE) water model [47] was used to fill the remaining box volume. The total number of the atoms in each simulation system was 30,209, 36,696, 25,048 including complex and waters.

In the simulation process, the whole system was first minimized by using 5000 steepest descent steps. This was followed by a 200 ps MD run to equilibrate the water molecules, protein and counterions. The simulation was run with LINCS algorithms [48] on hydrogen atoms and then continued for 5 ns by using 2 fs time step, where the coordinates were saved every 5 ps for analysis.

## 3. Results and Discussion

### 3.1. CoMFA and CoMSIA Statistical Results

An effective 3D-QSAR model is generated by considering a number of statistical parameters, such as the cross-validated correlation coefficient (*r*^2^_cv_), non-cross-validated correlation coefficient (*r*^2^_ncv_), standard error estimate (SEE) and *F*-statistic values (*F*). The CoMFA and CoMSIA models are built after model development and validation based on internal predictions of the training set and the external predictions of the test set.

For CoMFA analysis, a cross-validated partial least-square (PLS) analysis is performed using the leave-one-out option which produces the cross-validated correlation coefficient (*q*^2^) values and the optimal number of components. Generally, in 3D-QSAR studies, a cross-validated regression coefficient *q*^2^ being higher than 0.5 can be considered as statistical proof with high predictability [49]. Then, using the optimal number of components found earlier, the non-cross-validated PLS analysis is performed to produce a non-cross-validated correlation coefficient (*r*^2^_ncv_), a standard error estimate (SEE) and *F*-statistic values which can combine to evaluate the models.

For CoMSIA analysis, five descriptors (steric, electrostatic, hydrophobic, hydrogen-bond-donor and hydrogen-bond-acceptor) are considered. However, some previous work has found that the five descriptor fields are not totally independent of each other and such dependencies of individual fields might decrease the statistical significance of the models [50,51]. Therefore, in this work all 31 possible descriptors’ combinations for each group are calculated to provide the optimal models. The statistical results are summarized in Table 1.

In both the CoMFA and CoMSIA analysis, the models produced from the ligand-based alignment are better than the receptor-based alignment. So, we mainly discuss the ligand-based models in this study. Other than the optimum results listed in this paper, the others are shown in the supporting information (Tables S4–S9).

### 3.2. Validation of the 3D QSAR Models

**Figure 4.**
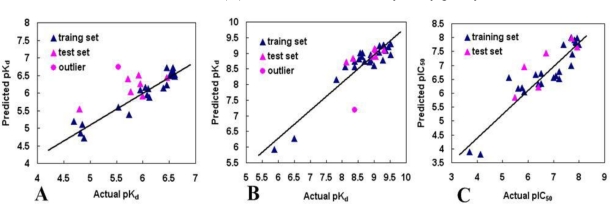
Plot of the predicted p*IC*_50_ *versus* the experimental p*IC*_50_ values for the models. (**A**) COMFA model of benzamide tetrahydro-4*H*-carbazol-4-one analogs; (**B**) CoMSIA model of AT13387 Derivatives; (**C**) CoMSIA model of dihydroxylphenyl amides.

A predictive correlation coefficient *r*^2^_pred_ is used to determine the stability and predictive abilities of the obtained models from the test sets which were not used to construct the models. The resultant optimum models exhibit agreeable statistical results for three classes and are shown in Table 1. The correlation between the CoMFA and CoMSIA predicted activities and the experimental activities of the test set compounds are shown in Figure 4. The test results indicate that the CoMFA and CoMSIA models are reliable.

#### 3.2.1. BT

Eight compounds (shown in Table S1) out of the total twenty eight Hsp90 inhibitors were used as the test set and the remaining compounds were used as the training set. The statistical results of the optimal model are shown in Table 1. The optimal CoMFA model gives a *q*^2^ of 0.428 for two components and *F* of 78.818, with a *SEE* of 0.22 and shows good predictive ability. However, the CoMSIA model shows poor internal predictions (*r*^2^_cv_ = 0.408) relative to the CoMFA model using steric and electrostatic fields. Meanwhile, an incorporation of the hydrophobic field makes the models perform poorer, and attempting to use all possible field combinations also could not improve the model performance. All this suggests that the CoMFA model is superior to the CoMSIA model for this class of Hsp90 inhibitors.

For the results of CoMFA model, the *r*^2^_pred_ is 0.2769 before omitting compound 11; after discarding it, the predicted value increased to 0.5747, indicating compound 11 is the outlier. The outlier status of compound 11 can stem from its low activity (*K_d_* = 2.9), compared to its counterpart, compound 12. Furthermore, it has a higher residue between the observed and predicted biological activity which further confirms the robustness and statistical confidence of the derived model.

#### 3.2.2. AT

The statistical parameters of the optimal model, for AT, are summarized in Table 1. The highest *q*^2^ of 0.715 is obtained with two components. *F* = 86.941, *SEE* = 0.304, *r*^2^_pred_ = 0.7013 and *SEP* = 0.494 for the model derived from the combinations of SED descriptors. At the same time, the model derived from the combinations of SEHDA also shows comparable predictions. However, incorporation of hydrophobic and hydrogen-bond-acceptor fields to SED, led to no notable improvement in statistical features (*q*^2^ = 0.728, *SEE* = 0.268, *F* = 115.04, *r*^2^_pred_ = 0.5398). So, the model shown in Table 1was selected as the optimum model for further analysis, showing reasonable statistical features. On the other hand, the CoMFA model gives a *q*^2^ of 0.604 for two components, *r*^2^_pred_ of 0.7272. Overall, the performance of the CoMSIA model is superior to that of the CoMFA one. The decrease of contribution of steric field in the CoMSIA model (shown in Table 1) can be explained by introducing a hydrophobic field in the CoMSIA model, and also by the fact that in some cases, the steric field is correlated to some extent with hydrophobic field [52].

Compound 6 in the CoMFA and CoMSIA models was treated as an outlier. On inclusion of this compound, the model showed poor predictive ability with *r*^2^_pred_ of 0.2225. Omission of compound 6 resulted in an increased *r*^2^_pred_ value of 0.7272 for CoMFA model. There are several reasons that may account for outliers, including unique structural differences, different binding conformations, and a higher residual between the observed and predicted biological activity of an inhibitor. Compound 6 was very similar in structure to compound 5; the only difference being that the substituent of compound 6 is a cyclopropyl, which is also the only inhibitor with cyclic substituent in this position, and might account for its outlier status.

#### 3.2.3. DA

In this model, the data set is divided into a training set of 23 and a test set of 6 molecules. The most active compound, 19, was selected as a template to sketch the rest of the molecules. Figure 2C shows the aligned molecules which are further used to generate the CoMFA and CoMSIA columns. The CoMSIA results are shown in Table 1. The CoMFA and CoMSIA statistical data show *q*^2^ of 0.401 for CoMFA, and 0.645 for the CoMSIA models, respectively, indicating that the CoMSIA model has better internal predictive ability than the CoMFA model.

In the CoMSIA model, PLS analysis of this group of Hsp90 inhibitors of the training set showed cross-validated *q*^2^ of 0.645 using two principal components, and the non cross-validated *r*^2^ value was 0.858, with a *SEE*, *SEP* and *F* value of 0.478, 0.757 and 60.608, respectively. The CoMFA model exhibits a *q*^2^ of 0.401 with two compounds, with the non-cross-validated *r*^2^ of 0.724, *F* = 26.192 and *SEE* of 0.668. Table 1 shows that the steric field and electrostatic field have an almost similar influence on producing the CoMFA model. This 3D-QSAR model was further validated using the external test set. Both the CoMFA and CoMSIA models gave *r*^2^_pred_ of 0.6913 and 0.7177, respectively. The values suggest that all these models have good generalization ability in the test set.

### 3.3. 3D-QSAR Contour Maps

CoMFA, and CoMSIA contour maps were generated to visualize the information of the derived 3D-QSAR model by plotting the coefficients from CoMFA and CoMSIA QSAR models, which represent the lattice points and the difference in the field values at lattice point. Briefly, the contour maps indicate regions in 3D space around the molecules where changes in the particular physicochemical properties are predicted to increase or decrease potency. To aid in visualization, the most active compounds are shown with the contour maps.

#### 3.3.1. BT

**Figure 5.**
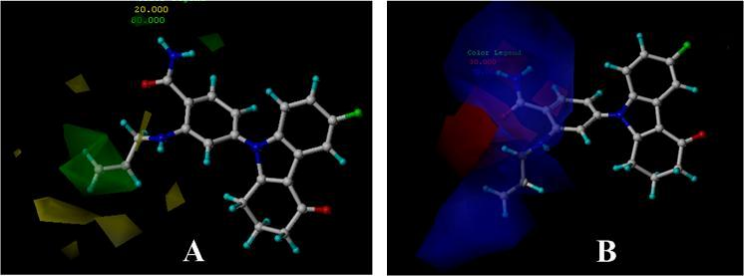
CoMFA StDev*Coeff contour plots for benzamide tetrahydro-4*H*-carbazol-4-one analogs in combination with compound 17. (**A**) The green (sterically favorable) and yellow (sterically unfavorable) contours represent 80% and 20% level contributions, respectively; (**B**) The blue (electropositive charge favorable) and red (electronegative charge favorable) contours represent 70% and 30% level contributions, respectively.

**Figure 6.**
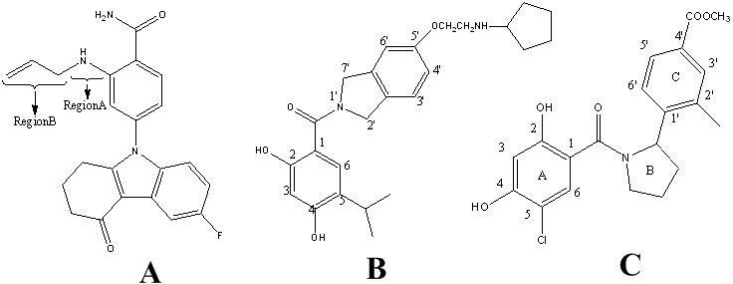
The most active molecule showing different regions which are used in contour analysis. (**A**) Compound 17 in the group of benzamide tetrahydro-4*H*-carbazol-4-one analogs; (**B**) Compound 24 in the class of AT13387 Derivatives; (**C**) Compound 19 in the series of dihydroxylphenyl amides.

The CoMFA contour plots of steric and electrostatic interactions are shown in Figure 5. Figure 5A shows the steric contour map with the highly active inhibitor 17 (p*K*_d_ = 6.6021) as a reference. A large region of green contour near Region B (shown in Figure 6A) indicates that steric bulk is favored there. This is consistent with the reported experimental results that compounds 7, 8, 9, 10 with bulky substituent were more active than compounds 1–6 with small substituents [32]. A small yellow contour is close to the N atom of Region A, which indicates that bulky groups at this position would decrease the potency. Several yellow contours are observed outside the Region B and around the green contour, which strongly delimits the sideward relocatability. For example, compounds 16, 17, 18 with high inhibitory activity can be explained as they have moderate substituents at the R1 position that cannot reach the yellow contour area. However, molecules 19, 20, 22, and 24 show complete incorporation of extended side chain into the yellow region, apparently responsible for their lower potency. This indicates that the optimal length of substituent at R1 position would enhance the activity of these compounds (10 and 17).

The electrostatic contour map is shown in Figure 5B. Two blue polyhedra encompassing the benzamide indicate positively charged groups which are beneficial to the activity. Positively charged propylene at Region B of compound 17 leads to its high activity (p*K*_d_ = 6.6021), while the negatively charged bromo, cyano and methoxy group at the same position of compounds 3, 4, 5 respectively show inactivity. Three red contours at the opposite sites of the blue polyhedra indicate that electronegative groups are tolerated at this position. Both of the electropositive favored and electronegative contours emerge at the same region which indicates that a balance of these properties among the groups present at this region is required for optimum binding.

#### 3.3.2. AT

**Figure 7.**
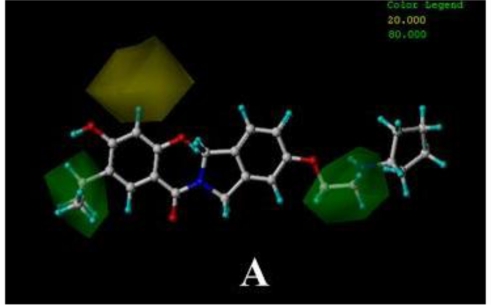

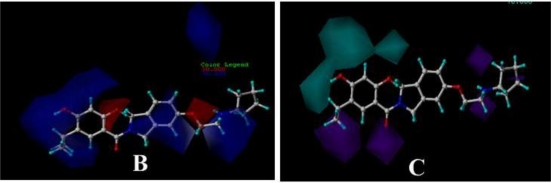
CoMSIA StDev*Coeff contour plots for AT13387 Derivatives in combinition with compound 24. (**A**) The green (sterically favorable) and yellow (sterically unfavorable) contours represent 80% and 20% level contributions, respectively; (**B**) The blue (electropositive charge favorable) and red (electronegative charge favorable) contours represent 70% and 30% level contributions, respectively; (**C**) The cyan (hydrogen-bond-donor favorable) and purple (hydrogen-bond-donor unfavorable) contours represent 70% and 30% level contributions, respectively.

The contour maps of CoMSIA (steric, electrostatic, hydrogen-bond-donor) are represented by color codes shown in Figure 7. To aid in visualization, the highly active inhibitor 24 was overlaid in the maps.

Figure 7A shows the CoMSIA steric field. One medium sized of green contour near the 5′ position (shown in Figure 6B) of benzamide ring indicates that bulk subtituents are favorable in this region. Another green contour region, around the 5-position of isoindoline, suggests that a bulky substituent is preferred in this position to produce higher inhibitory activity. The activity of compound 12 decreased after the −F substituent on that position was replaced with a less bulky group, such as compound 10 (−Cl). The yellow contour near the 3-position of benzamide ring indicates the need for a small substituent in this area to enhance the biological activity. This is consistent with the reported experimental results of one compound which is not listed in this study because it binds very weakly [33].

The graphical interpretation of the electrostatic interaction in the CoMSIA model is represented in Figure 7B. The red contour near the 5′ position of isoindoline indicates electronegative groups are beneficial to the activity. This is in accord with compounds 23, 24, 25 and compounds 17, 31, 32, the activity of the former is larger than that of the latter, because the former possesses a more electronegative atom (−O) than the latter (−C) at this position. Another red contour appears at the 2-position of benzamide, showing that an electronegative group here is important for inhibitory activity. The blue contour seperate from the main contour map indicates that electropositive groups which can extend to this region add to its activity. The large blue contour around the isoindoline suggests electropositive groups are preferred at this position. The electronegative oxygen atom at 4-position of benzamide embedded in the blue contour, suggests that it is unfavorable for the inhibitory activity. Therefore, we suggest replacing the hydroxyl with an electropositive group, which may be beneficial to the activity.

Figure 7C indicates areas where hydrogen-bond-donors in the ligand promote or decrease the inhibitory activity. Two purple contours either side of the R4 substituent suggest that H-bond donor groups are disfavored here; this can be explained by compound 29 with two -NH in this region exhibiting lower activity (p*K*_d_ = 8.8861) than compound 24. However, the most potent compound 24 also has one H-bond donor group −NH in this area suggesting that there is room for modification at this position considering the effect of H-bond donor. The large polyhedra around the benzamide implies that H-bond donor groups could have a positive effect on the inhibitory activity. In addition, it has H atom at the 3-position. From the contour, we can conclude that maybe it is better for the inhibitory activity if the H atom is transformed to other H-bond donor groups.

#### 3.3.3. DA

**Figure 8.**
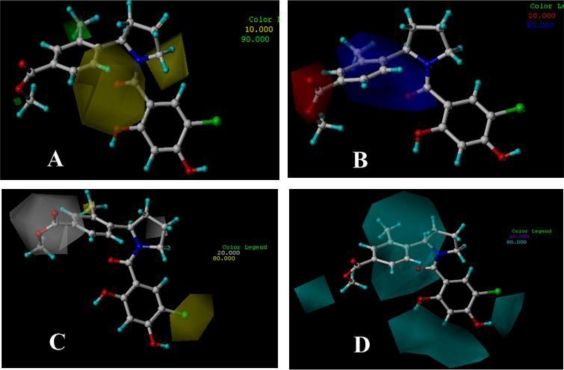
CoMSIA StDev*Coeff contour plots for Dihydroxylphenyl amides in combination with compound 19. (**A**) The green (sterically favorable) and yellow (sterically unfavorable) contours represent 90% and 10% level contributions, respectively; (**B**) The blue (electropositive charge favorable) and red (electronegative charge favorable) contours represent 80% and 20% level contributions, respectively; (**C**) The yellow (hydrophobic favorable) and white (hydrophobic unfavorable) contours represent 80% and 20% level contributions, respectively; (**D**) The cyan (hydrogen-bond-donor favorable) and purple (hydrogen-bond-donor unfavorable) contours represent 80% and 20% level contributions, respectively.

The CoMSIA contour maps of steric, electrostatic, hydrophobic and hydrogen-bond-donor fields are shown in Figure 8. The most potent compound, 19, is displayed as a reference.

Figure 8A depicts the CoMSIA steric contour map of the optimal QSAR model. A small green contour located on the outer side of C4′ position of ring C (shown in Figure 6C) represents regions where bulky groups can increase activity. In addition, another sterically favorable green contour, near the C2′ position of ring C, suggests that maybe substituents larger than methyl in this region is good for increasing the activity; therefore, reasonable structural modifications can be carried out to improve the activity and selectivity of Hsp90 inhibitors. The medium sized yellow contour localizes at the linker area between ring B and ring C indicating bulkier groups are unfavored at this position; for example, compounds 6 and 8 have bulkier linker (−SO_2^−^_, −COOCH_2^−^_) in this region which also have lower inhibitory activity than compound 19.

The CoMSIA electrostatic contour map is presented in Figure 8B. The red contour overlapping the −COOCH_3_ group of compound 19 means that this group is favorable in this region and will lead to enhanced activity. The large blue contour encompasses ring A and the linker between ring B and ring C, denotes the region where electropositive groups are preferred in this region; for example, the activity of compounds 11 and 12 with the Cl^−^ and CH3O^−^ are less potent than compounds 14 which possesses H− at this position.

The hydrophobic contour map of a CoMSIA model is shown in Figure 8C. The large yellow region at the C5 position of ring A stresses that hydrophobic substitutions will increase the inhibitor affinity. Due to another small yellow contour at the C2′ position of ring C, because of hydrophobic −CH_3_ substituent at C2′ position, compound 19 displays high activity; whereas for the weak compounds, 9–15, no such substituent is present. Besides, at C4′ position of ring C, there is a large white contour, suggesting that hydrophilic groups in this area are favorable for inhibitory activity.

Figure 8D shows the CoMSIA H-bond donor contour map. For this class of inhibitors, there are indeed less H-bond donor groups, therefore, the contour map here is mainly used for considering further modification of these compounds. A medium sized and a small cyan contour, around ring A, suggests that H-bond donor substituents are favorable for activity. However, at C5 and C3 position of ring A, there are chlorine and hydrogen which indicate that incorporation of H-bond donor groups at these two positions may improve the inhibitory activity. One small H-bond donor unfavorable purple contour, which is encompassed by the cyan contour, suggests the C atom in this area is very important to the inhibitory activity. That is, modification on ring C may be useful and a requirement to increase activity.

### 3.4. Docking Analysis and Comparison with 3D Contour Maps

Other than ligand-based 3D-QSAR analysis, we also performed receptor-based 3D-QSAR studies, which not only provides the natural interaction between the ligands and the receptor, but also applies for 3D-QSAR studies. In order to ensure the validity of docking calculations and of the conditions and parameters of docking, the ligands were flexibly redocked to the binding site of Hsp90 protein and the optimum docking conformations as the most probable binding conformation corresponding with the lowest energy score. The low RMSD values for the three different classes of inhibitors are 0.17 Å, 0.05 Å and 0.02 Å, respectively. This means that the docking procedure is reliable and will reproduce the receptor bound conformation of other molecules in the dataset.

Lastly, we analyzed whether the docking scores can be correlated to the biological data of the inhibitors. At the same time, we tried to compare the docking results with the 3D contour maps we analyzed above.

#### 3.4.1. BT

**Figure 9.**
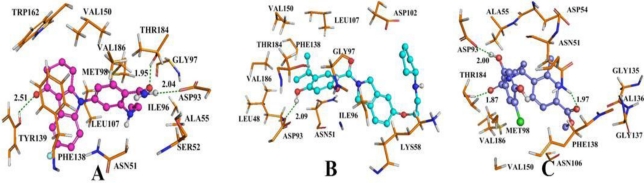
The enlargement for the structure of the binding site with substrates which are displayed in ball and sticks, H-bonds are shown as dotted green lines, and the nonpolar hydrogens were removed for clarity. (**A**) Docked conformation derived for compound 17 in complex to the active site of Hsp90 enzyme; (**B**) Docked conformation derived for compound 24 with the binding site of Hsp90; (**C**) The docking structure of compound 19 with the allosteric binding site of Hsp90 enzyme.

The binding mode of the most active compound, 17 shown in Figure 9A, is taken as an example for analysis. The ligand-receptor interaction results show that Met98, Ile96, Ala55, Thr184, Asp93, Asn51, Phe138, Tyr139, Leu107, Trp162 are the important residues at the active site and are the main contributors to the ligand and receptor interaction. The amide oxygen at the para position of benzamide ring forms hydrogen bond with OH of Thr184 (−O···HO, 1.95 Å, 130.2°). The oxygen substituent of carbazol acts as an acceptor to form H-bond with OH of Tyr139 (−O···HO, 2.51 Å, 114.6°). Another H-bond formed between the amino hydrogen at the para position of benzamide and the oxygen atom of Asp93 (−O···HN, 2.04 Å, 156°). These results coincide with the previously quoted paper [32]. Residues Ala55, Ile96, Met98 formed hydrophobic contacts with the ligands which are favorable for the inhibitory activity. From Figure 9A, we can see the propylene substituent partially extended outside the binding pocket, as well as, the steric contour map (shown in Figure 5A) had a green contour at this position. On the other hand, the carbazol group forms π-π bond with Phe 138 which further enhanced the interaction between the ligand and the receptor.

#### 3.4.2. AT

Compound 24 was selected as a reference to analyze the binding mode of docking shown in. Figure 9B From the map, we can see that the −OH group at C4 position of benzamide acts as a H-bond donor to form hydrogen bond with oxygen atom of carboxyl of Asp93 (−O···HO, 2.09 Å, 145.7°), which matched well with the H-bond donor contour depicted in Figure 7C. There is a large cyan contour around C4 position of benzamide which indicates that H-bond donor groups in this area are favorable for the inhibitory activity. One hydrophobic pocket composed of residues Leu48, Leu107, Val150, Va186, and the steric contour map with a green contour at C5 position of benzamide, suggests that bulky substituents in this area can increase the activity. However, large bulky substituents will lead to steric clash with these hydrophobic amino acids in this area, and would result in decreasing activity. Therefore, it is not benefitial to introduce too large groups to this position. At the same time, a group of hydrophilic amino acid Asp102, Lys58, Asn48 around C5′ position of isoindoline suggests that this area can hold bulky substituents, which coincides with the steric contour map depicted in Figure 7A. At the docking map, the subtituent at this position partially extended outside the binding pocket, which further indicates that bulky groups are favorable in this area for the inhibitory activity. The electropositive favorable blue contour at the C5 position of benzamide ring is surrounded by Thr184, Asp93, Asn51, which can validate the 3D contour map analysis that the activity will be enhanced by introducing electropositive groups at this position. In addition, another blue contour at C5′ position of isoindoline indicates electropositive substituents are favorable, which is consistent with the docking result whereby it favorably interacted with the backbone −NH of Asn106 and the −OH of Asp102.

#### 3.4.3. DA

Figure 9C shows the result of the docking study with compound 19 as a template. Compound 19 forms a hydrogen bond between the oxygen atom of the −COO group at 4′ position of ring C and hydrogen atom of −NH group of Asn51 (−O···HN, 1.97 Å, 142.4°). At the same time, the carbonyl oxygen atom at the linker, between ring A and ring B, forms hydrogen bond with the hydrogen atom of −OH group of Thr184 (−O···HO, 1.87 Å, 154°). In addition, the third hydrogen bond forms between hydrogen atom of −OH group which is at C2 position of ring A and the oxygen atom of the −COO group of Asp93 (−O···HO, 2.00 Å, 114.4°). This is consistent with the H-bond donor contour map (Figure 8D). The cyan contour around the C2 position of ring A suggests this is a H-bond donor favorable region. All of the hydrogen bonds described above are the same as those described in the original paper [34]. Figure 9C displays the methyl substituent at C2′ position of ring C embedded into a relatively large pocket composed of Val136, Gly135, Asn106. From the docking results, we can see that there is large free space in this pocket, suggesting that there is sufficient room for introducing bulky substituents, which is also validated by a sterically favorable green contour at this position seen in the CoMSIA model. The hydrophilic favorable white contour at the C2′ position of ring C is enclosed by Asn106, Asn51 and Gly137, which explains the increased activity after introducing hydrophilic groups in this area. The C2′ position of ring C is surrounded by carbonyl group of Asp54 and Ser50 suggesting that electropositive substituents in this area are favorable for a binding activity of a ligand, correlating well with the analysis of the electrostatic contour map of the CoMSIA model which has a blue contour at this position. The red contour (Figure 8B) at the C4′ position of ring C suggests an electronegative favorable region, corroborated by introducing suitable electronegative groups which would have a favorable interaction with the backbone NH of Phe138 and Asn51.

That is, the results of the docking studies and 3D-QSAR can complement and validate each other, indicating that the 3D-QSAR model which we developed is reasonable and can offer constructive suggestions to the further rectification and modification of inhibitors of Hsp90.

### 3.5. Comparison of Binding Modes for Each Class

**Figure 10.**
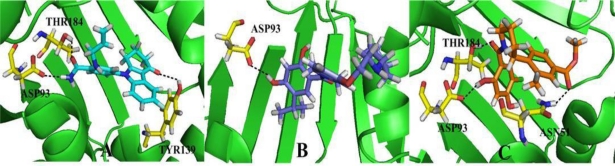
Docked conformations derived from compounds 17, 24, 19 with the binding site of Hsp90 protein; Hydrogen bonds are shown by broken lines; Compounds 17, 24, 19 are shown in A, B and C, respectively as a reference, and are depicted in different colors: cyan, blue and orange. Thr184, Asp93, Tyr139, Asn51, as the significant residues, are colored by atom type (C, yellow; N, blue; H, white; O, red).

From the docking results, we can compare the inhibitory activity of the three series of Hsp90 inhibitors in order to better understand the variations in biological activities. According to the docking study, for BT, it mainly formed three hydrogen bonds between compound 17 and Thr184 (−O···HO, 1.95Å, 130.2°), Tyr139 (−O···HO, 2.51 Å, 114.6°) and Asp93 (−O···HN, 2.04 Å, 156°) of the receptor (Figure 9A). Additionally, for AT, only one hydrogen bond formed during the docking procedure. Asp93 (−O···HO, 2.09 Å, 145.7°) served as the hydrogen bond donor to form hydrogen bond with compound 24 (Figure 9B). As for the class of DA, three hydrogen bonds were formed. From Figure 9C, we can see that Thr184 (−O···HO, 1.87 Å, 154°), Asn51 (−O···HN, 1.97 Å, 142.4°) and Asp93 (−O···HO, 2.00 Å, 114.4°) were involved in the hydrogen bonding contacts with compound 19. From the docking study, we can simply conclude that the three series of Hsp90 inhibitors all involve one common active amino acid residue Asp93 (see Figure 10). On the other hand, BT and DA all form three hydrogen bonds which are more than the AT. Thus, we can assume that the activities of BT and DA are higher than AT. On the other hand, due to the fact that the docking analysis in this work eliminates the effect of environment, we do not consider waters which can form hydrogen bonds with inhibitors to enhance its stability and inhibitory activity. Therefore, it needs further analysis to validate the docking results.

### 3.6. Molecular Dynamics Simulations

When predicting the binding orientation of ligand candidates to the binding site, docking analysis can provide a starting point for further calculations for the purpose of predicting the binding affinity of the molecules and to evaluate the stability of the predicted interactions involved in binding. In turn, MD simulations were undertaken to consider the impacts of the receptor flexibility and the effects of water solvation on the complex.

The three docked complex structures of 3D0B with ligand 17 (the most potent compound of BT), 2XJG with ligand 24 (the most potent compound of AT), 3K97 with ligand 19 (the most potent ligand of DA) have done a 5 ns simulation. The simulations were performed to obtain a dynamical picture of the conformational change in the MD simulations; the main purpose is to explore the conformational alterations that take place in the ligand and the receptor.

#### 3.6.1. BT

**Figure 11.**
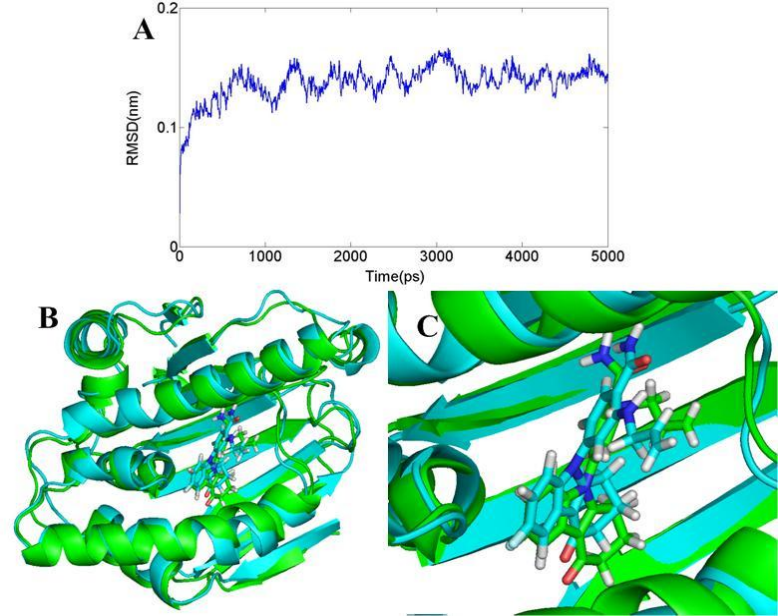
(**A**) Plot of the root-mean-square deviation (RMSD) of docked complex *versus* the MD simulation time in the MD-simulated structures; (**B**), (**C**) View of superimposed backbone atoms of the lowest energy structure of the MD simulation (cyan) and the initial structure (green) for compound 17-3D0B complex. Compound 17 is represented as carbon-chain in green for the initial complex and carbon-chain in cyan for the lowest energy complex.

**Figure 12.**
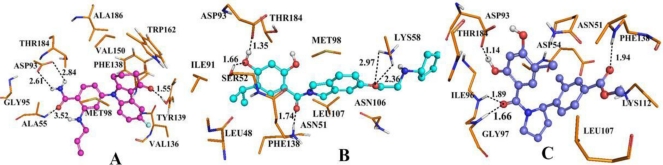
Plot of the MD-simulated structures of the binding site with ligand. H-bonds are shown as dotted black lines; Active site amino acid residues are represented as sticks; the inhibitors are shown as stick and ball model. (**A**) Compound 17 in complex to the active site of Hsp90 enzyme; (**B**) Compound 24 with the binding site of Hsp90; (**C**) Compound 19 with the allosteric binding site of Hsp90 enzyme.

The RMSD of 3D0B complex is shown in Figure 11A. The RMSDs of the trajectory with respect to their initial structure range from 1.2 to 1.5 Å. After 2 ns, the RMSDs of the complex reach about 1.4 Å, and retain this value throughout the simulation. A superposition of the stable structure in all the MD simulations (coming after 2 ns) and the initial docked structure is shown in Figure 11B, C. After MD simulation, we analyzed interactions between compound 17 and the receptor in order to explore the similarity and difference between the results of docking and MD simulation. From the average structure extracted from the MD simulation, we can see that it mainly forms four hydrogen bonds (shown in Figure 12A). A proper H-bond (1.55 Å) is built between the oxygen substituent of carbazol and Tyr139; the docked model of the complex also formed hydrogen bond with Tyr139 with a distance of 2.51 Å, which is bigger than the former. The other two hydrogen bonds formed between the two amino hydrogens at the para position of benzamide ring and Asp93 (−O···HN, 2.61 Å, 99.6°), (−O···HN, 2.84 Å, 106.1°). For the docking result, only one hydrogen bond was formed in this position. In addition, the amide oxygen atom at the para position of benzamide ring acts as hydrogen acceptor to form H-bond with the amino group N atom of Ala55 (−O···HN, 3.52 Å, 100.4°). At the same time, it also produces π-π bond between the carbazol and Phe138 to enhance its stable conformation and binding activity.

#### 3.6.2. AT

**Figure 13.**
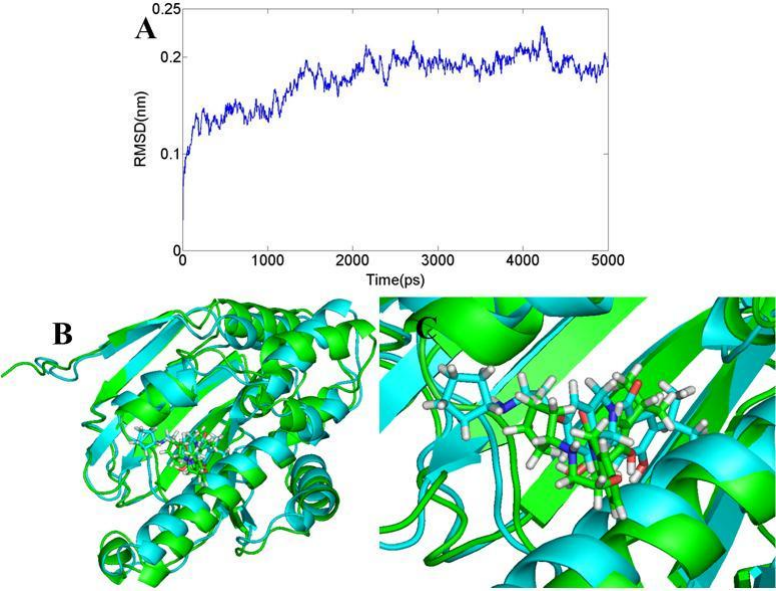
(**A**) Plot of the root-mean-square deviation (RMSD) of docked complex *versus* the MD simulation time in the MD-simulated structures; (**B**), (**C**) View of superimposed backbone atoms of the lowest energy structure of the MD simulation (cyan) and the initial structure (green) for compound 24-2XJG complex. Compound 24 is represented as carbon-chain in green for the initial complex and carbon-chain in cyan for the lowest energy complex.

As for this class of inhibitors, the RMSDs of the trajectory with respect to their initial structure ranging from 1.3 to 2.2 Å are depicted in Figure 13A. A superposition of the average structure of ensemble and the docked structure is shown in Figure 13B. Figure 12B shows the conformation derived for compound 24 with the allosteric binding site of 2XJG, in which five hydrogen bonds were produced which is more than the docking process. The hydroxyl group H atom at C4 position of benzamide forms H-bond with the oxygen atom of carboxyl of Asp93 (−O···HO, 1.35 Å, 165.1°) as well as the docking result. However, the hydrogen bond formed by the docking process with a distance of 2.09 Å. The other H-bonds form between the hydroxyl group H atom at C4 position of benzamide and the oxygen atom of Ser52 (−O···HO, 1.66 Å, 139.9°); the oxygen atom of carboxyl between the benzamide ring and the isoindoline ring forms hydrogen bond with the Asn51 (−O···HN, 1.74 Å, 113.5°). The oxygen atom of the substituent at the C5′ position of isoindoline forms two H-bonds with the backbone NH_2_ of Lys58 (−O···HN, 2.97 Å, 80.2°) and (−O···HN, 2.36 Å, 116.9°).

#### 3.6.3. DA

**Figure 14.**
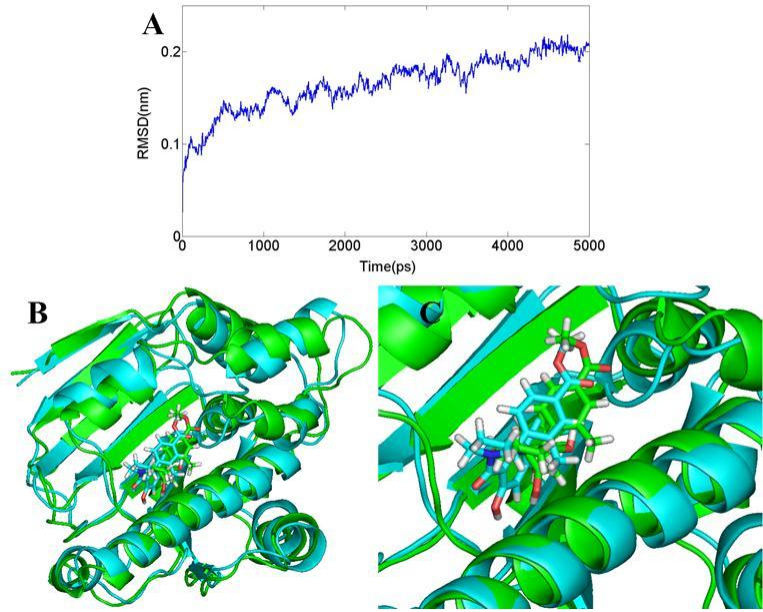
(**A**) Plot of the root-mean-square deviation (RMSD) of docked complex *versus* the MD simulation time in the MD-simulated structures; (**B**), (**C**) View of superimposed backbone atoms of the lowest energy structure of the MD simulation (cyan) and the initial structure (green) for compound 19-3K97 complex. Compound 19 is represented as carbon-chain in green for the initial complex and carbon-chain in cyan for the lowest energy complex.

Figure 14A shows the RMSD values, the RMSDs of the trajectory with respect to their initial structure ranging from 1.4 to 2 Å for 3K97. After 2 ns, the RMSDs of the complex reach about 1.8 Å and retain these values throughout the simulation. This clearly indicates that the docked complexes can reach metastable conformation after 2 ns of simulation. Figure 14B, C represents the superimposed backbone atoms of the lowest energy structure of the MD simulation and the initial structure. By combining Figure 14B, C and Figure 12C, we attempt to analyze the differences between the structure extracted from MD simulations and the docked model of the complex. The docked complex structure of 3K97 with ligand 19 was further used for molecular dynamics to obtain a dynamical picture of the conformational changes. Figure 12C shows that the ligand core is anchored in the binding site via four H-bonds. The hydrogen atom of −OH group at C2 position of ring A interacts through H-bonding with the backbone −COO of Asp93 (−O···HO, 1.14 Å, 133.4°), and the same H-bond is formed for the docking result, but with a distance of 2.00 Å. In addition, the carbonyl oxygen atom at the linker between ring A and ring B form two hydrogen bonds with NH group of Gly97 (−O···HN, 1.66 Å, 167.2°) and Ile96 (−O···HN, 1.89 Å, 137.6°). Also the oxygen atom of the COO− group at C4′ position of ring C forms H-bond with NH group of Phe138 (−O···HN, 1.94 Å, 165.5°). All these three hydrogen bonds are different from the docking results.

The analysis described above suggest that there is no significant differences between the stable structure extracted from MD simulations and the docked model of the complex, which indicates that the docking model is rational and valid. But there are also some differences between them, such as the number and length of hydrogen bonds and the main amino acids involved in forming hydrogen bonds. To sum it up, the conformations obtained after molecular dynamics are more stable than the docked conformations. This coincides with the QSAR results derived from the receptor-based method, which have poorer prediction than the ligand-based method. This also can be explained by the fact that the docking method itself has some shortcomings and does not consider the impact of solvents which are not explicitly treated. However, MD simulation is performed in a more realistic environment, and much closer to physiological conditions. In addition, compared with the docking analysis, the corresponding binding mode from MD simulation has better correlation with the 3D-QSAR analysis.

## 4. Conclusions

3D-QSAR studies on three kinds of Hsp90 inhibitors using CoMFA and CoMSIA based on ligand- and receptor-based methods have led to the identification of important structural features of steric, electrostatic, hydrophobic and H-bond donor interactions between the receptor and its ligand. The obtained QSAR models are reliable, evidenced by the high *r*^2^_cv_ values and small standard errors, and the 3D contour maps are also well correlated with docking models.

For BT series, the CoMFA model yields relatively better prediction than the CoMSIA one, which reveals the importance of the steric effect, making a contribution of 82.5% to the model. The results indicate that −R1 group was very important for the inhibitory activity. Docking studies reveal that the amino hydrogen and the oxygen atom at the para position of benzamide, and the oxygen substituent of carbazol plays a main contribution to enhance the activity.

For AT derivatives, the optimal CoMSIA model shows that the bulky electropositive groups at C5 position of benzamide and electropositive; H-bond donor groups at positions C3, C4 of benzamide had a positive effect on the inhibitory activity. The docking study reveals that substituent at C5 position of benzamide can bind to a hydrophobic pocket, while the −OH group at C2 position of benzamide forms hydrogen bond with the backbone of Asp93.

For DA analogs, the contour map showed that the substituents at C2 and C4 position of ring C had a great impact on the overall inhibitory activities, which is consistent with the docking results. Electropositive substituent at C2 position is surrounded by Asp54 and Ser50, which was favorable for a binding activity. In addition, electronegative substituent at C4 position has a good interaction with the backbone NH of Phe138 and Asn51. It also formed hydrogen bond with hydrogen atom of −NH group of Asn51. In summary, good consistency between the 3D-QSAR, the docking and MD modeling results indicates the robustness of the 3D-QSAR models, which can serve as a basis for modification and design of novel anticancer compounds with enhanced activities.

## Supplementary Material

Supplementary material is available on the publisher’s website along with the published article.



## Figures and Tables

**Table 1.** The best results of the CoMFA and CoMSIA analyses for the training and test set compounds.

**Parameters**	**Benzamide tetrahydro-4*H*-carbazol-4-one analogs**	**AT13387**	**Dihydroxylphenyl amides**

	**CoMFA**	**CoMSIA**	**CoMSIA**
*R*^2^_cv_	0.482	0.715	0.645
*R*^2^_ncv_	0.903	0.892	0.858
*SEE*	0.22	0.304	0.478
*F*	78.818	86.941	60.608
*R*^2^_pred_	0.5747	0.7013	0.7177
*SEP*	0.507	0.494	0.757
*N*_c_	2	2	2

**Field Contribution**			

S	0.825	0.179	0.153
E	0.175	0.322	0.285
H	-	-	0.29
D	-	0.499	0.273
A	-	-	-

*R*^2^_cv_ = Cross-validated correlation coefficient after the leave-one-out procedure; *R*^2^_ncv_ = Non-cross-validated correlation coefficient; *SEE* = Standard error of estimate; *F* = Ratio of *R*^2^_ncv_ explained to unexplained = *R*^2^_ncv_/(1 − *R*^2^_ncv_); *R*^2^_pred_ = Predicted correlation coefficient for the test set of compounds; *SEP* = Standard error of prediction; *N*_c_ = Optimal number of principal components; S = Steric; E = Electrostatic; H = Hydrophobic; D = Hydrogen-bond-donor; A = Hydrogen-bond-acceptor.
