# Supplementary material for: Structural Determination of Three Different Series of Compounds as Hsp90 Inhibitors Using 3D-QSAR Modeling, Molecular Docking and Molecular Dynamics Methods
Source: Int J Mol Sci. 2011 Jan 30;12(2):946–70. doi: 10.3390/ijms12020946 (PMC3083683; doi:10.3390/ijms12020946)
Supplement: Supplementary file 1 [file ijms-12-00946-s001.doc]

**Supplementary Material**

**Table S1.** Representative Skeletons and Molecular Structures of benzamide tetrahydro-4*H*-carbazol-4-one analogs and Their Binding Affinity values (p*K*_d_).

|  | | | | | | | | | |
| --- | --- | --- | --- | --- | --- | --- | --- | --- | --- |
| **Compound** | **R1** | | **R2** | | **R3** | | **R4** | | **p*K*_d_ (μM)** |
| 1 | H | | Me | | Me | | H | | 6.1249 |
| 2 | H | | H | | H | | H | | 5.7447 |
| 3 | Br | | H | | H | | H | | 4.699 |
| 4 ^a^ |  | | H | | H | | H | | 4.7959 |
| 5 | –OMe | | H | | H | | H | | 4.8239 |
| 6 | –NH_2_ | | H | | H | | H | | 5.5376 |
| 7 |  | | Me | | Me | | H | | 6.4559 |
| 8 ^a^ |  | | Me | | H | | H | | 5.7696 |
| 9 ^a^ |  | | H | | H | | H | | 6.4559 |
| 10 |  | | H | | H | | F | | 6.6021 |
| 11^a^ |  | | Me | | H | | H | | 5.5376 |
| 12 |  | | H | | H | | H | | 6.5686 |
| 13 ^a^ |  | | H | | H | | H | | 6 |
| 14 |  | | H | | H | | H | | 6.5376 |
| 15 |  | | H | | H | | F | | 6.1079 |
| **Table S1.** *Cont.* | | | | | | | | | |
| 16 |  | | H | | H | | H | | 6.4559 |
| 17 |  | H | | H | | F | | 6.6021 | |
| 18 |  | H | | H | | H | | 6.5086 | |
| 29 |  | H | | H | | H | | 4.8539 | |
| 20 |  | H | | H | | F | | 5.9586 | |
| 21 |  | H | | H | | H | | 6.0862 | |
| 22 |  | H | | H | | H | | 4.8861 | |
| 23^a^ |  | H | | H | | F | | 5.9208 | |
| 24 ^a^ |  | H | | H | | F | | 5.9586 | |
| 25^a^ |  | H | | H | | H | | 5.7212 | |
| 26 |  | H | | H | | F | | 6.5686 | |
| 27 |  | H | | H | | H | | 6.0458 | |
| 28 |  | H | | H | | H | | 6.3872 | |

^a^ Molecules Belonged to the Test Set.

**Table S2.** Representative skeletons and molecular structures of AT13387 derivatives and their binding affinity values (p*K*_d_).

|  | | | | | |
| --- | --- | --- | --- | --- | --- |
| **Compound** | **R1** | **R2** | **R3** | **R4** | **p*K*_d_ (μM)** |
| 1 | OH |  | H | H | 9.2676 |
| 2 |  |  | H | H | 7.8239 |
| 3 | –OCH_2_C(CH_3_)_2_– | | H | H | 5.8861 |
| 4 | –NHCH_2_C(CH_3_)_2_– | | H | H | 6.5086 |
| 5 | OH |  | H | H | 8.699 |
| 6 | OH |  | H | H | 8.3979 |
| 7 | OH |  | H | H | 8.5229 |
| 8 ^a^ | OH |  | H | H | 9 |
| 9 | OH | Cl | H | H | 8.3979 |
| 10 | OH |  | H | Cl | 8.6198 |
| 11^a^ | OH |  | F | H | 8.3372 |
| 12^a^ | OH |  | H | F | 9.5086 |
| 13 | OH |  | H |  | 8.5686 |

**Table S2.** *Cont.*

| 14 | OH |  | H |  | 9.0458 |
| --- | --- | --- | --- | --- | --- |
| 15 | OH |  | H |  | 9.301 |
| 16 | OH |  | H |  | 9.2924 |
| 17^a^ | OH |  | H |  | 8.9208 |
| 18 | OH |  | H | –O(CH_2_)_3_Morph | 9.1675 |
| 19 | OH |  |  | H | 8.1367 |
| 20^a^ | OH |  | –O(CH_2_)_3_Morph | H | 8.1024 |
| 21 | OH |  |  | H | 8.8539 |
| 22 | OH |  | H | –O-(CH_2_)_2_NH_2_ | 8.3979 |
| 23 | OH |  | H | –O-(CH_2_)_3_NH_2_ | 9.4437 |
| 24 | OH |  | H | –O(CH_2_)_2_NH-c-  Pentyl | 9.5229 |
| 25^a^ | OH |  | H | –O(CH_2_)_2_NH-neo-  Pentyl | 9.301 |
| 26 | OH |  | H |  | 8.6383 |
| 27^a^ | OH |  | H |  | 9.0223 |
| 28^a^ | OH |  | H |  | 9.0458 |

**Table S2.** *Cont.*

| 29 | OH |  | H |  | 8.8861 |
| --- | --- | --- | --- | --- | --- |
| 30 | OH |  | H |  | 9.3665 |
| 31 | OH |  | H |  | 8.8861 |
| 32 | OH |  | H |  | 9.1487 |

^a^ Molecules Belonged to the Test Set.

**Table S3.** Representative skeletons and molecular structures of dihydroxylphenyl amides and their binding affinity values (p*IC*_50_).

| (**a**) | | | | |
| --- | --- | --- | --- | --- |
| **Compound** | **Template** | **R1** | **X** | **p*IC*_50_(μM)** |
| 1 ^a^ | a |  | OH | 6.3979 |
| 2 | a |  | H | 5.7696 |
| 3 | a |  | H | 3.699 |
| 4 | a |  | H | 4.1308 |
| 5 ^a^ | a |  | H | 5.4949 |
| 6 | a |  | H | 5.8539 |
| 7 | a |  | H | 7.2218 |

**Table S3.** *Cont.*

| 8 | a | |  | | | H | | 6.301 | |
| --- | --- | --- | --- | --- | --- | --- | --- | --- | --- |
| 9 | a | |  | | | H | | 6.5229 | |
| 10 | a | |  | | | H | | 7 | |
| 11 | a | |  | | | H | | 7.0969 | |
| 12 | a | |  | | | H | | 6.3979 | |
| 13 | a | |  | | | H | | 5.6198 | |
| 14 | a | |  | | | H | | 7.2218 | |
| 15 | a | |  | | | H | | 5.2518 | |
| (**b**) | | | | | | | | | |
| **Compound** | | **Template** | | **R2** | **R3** | | **R4** | | **p*IC*_50_(μM)** |
| 16 | | b | | H | H | | H | | 7.699 |
| 17 | | b | | H | H | | COOCH_3_ | | 6.5229 |
| 18^a^ | | b | | H | H | | CONHCH_2_CH_3_ | | 5.8539 |

**Table S3.** *Cont.*

| 19 | b | | H | | COOCH_3_ | | H | 8 | |
| --- | --- | --- | --- | --- | --- | --- | --- | --- | --- |
| 20^a^ | b | | H | | CON(CH_3_)_2_ | | H | 7.9208 | |
| 21 | b | | H | | CONHCH_2_CH_3_ | | H | 7.3979 | |
| 22 | b | | COOCH_3_ | | H | | H | 7.7447 | |
| 23 ^a^ | b | | CON(CH_3_)_2_ | | H | | H | 6.699 | |
| (**c**) | | | | | | | | | |
| **Compound** | | **Chirality** | | **Template** | | **R5** | | | **p*IC*_50_(μM)** |
| 24 | | R/S | | c | |  | | | 7.9208 |
| 25 | | R/S | | c | |  | | | 7.8239 |
| 26 ^a^ | | R/S | | c | |  | | | 7.7212 |
| 27 | | R/S | | c | |  | | | 7.699 |
| 28 | | R/S | | c | |  | | | 7.8861 |
| 29 | | R/S | | c | |  | | | 7.8539 |

**Table S4. Summary of QSAR results of ligand-based model for benzamide tetrahydro-4*H*-carbazol-4-one analogs.**

|  | **CoMFA** | **CoMSIA** | | | | | | | |
| --- | --- | --- | --- | --- | --- | --- | --- | --- | --- |
|  | **SE** | **SE** | **SEH** | **SED** | **SEA** | **SEHD** | **SEHA** | **SEDA** | **SEHDA** |
| *R*^2^_cv_ | 0.428 | 0.408 | 0.339 | 0.294 | 0.226 | 0.334 | 0.252 | 0.295 | 0.336 |
| *R*^2^_ncv_ | 0.903 | 0.87 | 0.78 | 0.716 | 0.714 | 0.784 | 0.688 | 0.675 | 0.72 |
| *SEE* | 0.22 | 0.271 | 0.33 | 0.375 | 0.388 | 0.327 | 0.393 | 0.402 | 0.373 |
| *F* | 78.818 | 24.998 | 30.216 | 21.396 | 13.286 | 30.923 | 18.767 | 17.62 | 21.843 |
| *R*^2^_pred_ | 0.5747 | 0.5644 | 0.3585 | 0.6587 | 0.4255 | 0.3894 | 0.1654 | 0.3184 | 0.2763 |
| *SEP* | 0.507 | 0.577 | 0.572 | 0.591 | 0.638 | 0.574 | 0.609 | 0.591 | 0.573 |
| Nc | 2 | 4 | 2 | 2 | 3 | 2 | 2 | 2 | 2 |
| **Field Contribution** | | | | | | | | | |
| S | 0.825 | 0.498 | 0.232 | 0.148 | 0.251 | 0.106 | 0.139 | 0.094 | 0.076 |
| E | 0.175 | 0.502 | 0.265 | 0.179 | 0.256 | 0.125 | 0.159 | 0.117 | 0.092 |
| H | - | - | 0.504 | - | - | 0.295 | 0.332 | - | 0.203 |
| D | - | - | - | 0.673 | - | 0.474 | - | 0.511 | 0.41 |
| A | - | - | - | - | 0.493 | - | 0.369 | 0.279 | 0.218 |

*R*^2^_cv_ = Cross-validated correlation coefficient after the leave-one-out procedure; *R*^2^_ncv_ = Non-cross-validated correlation coefficient; *SEE* = Standard error of estimate; *F =* Ratio of *R*^2^_ncv_ explained to unexplained = *R*^2^_ncv_/(1 − *R*^2^_ncv_); *R*^2^_pred_ = Predicted correlation coefficient for the test set of compounds; *SEP*= Standard error of prediction; Nc = Optimal number of principal components. S = steric, E = electrostatic, H = hydrophobic, D = H-bond donor, A = H-bond acceptor.

**Table S5. Summary of QSAR results of receptor-based model for benzamide tetrahydro-4*H*-carbazol-4-one analogs.**

|  | **CoMFA** | **CoMSIA** | | | | | | | |
| --- | --- | --- | --- | --- | --- | --- | --- | --- | --- |
|  | **SE** | **SE** | **SEH** | **SED** | **SEA** | **SEHD** | **SEHA** | **SEDA** | **SEHDA** |
| *R*^2^_cv_ | 0.427 | 0.352 | 0.44 | 0.303 | 0.275 | 0.351 | 0.411 | 0.265 | 0.306 |
| *R*^2^_ncv_ | 0.96 | 0.51 | 0.967 | 0.512 | 0.451 | 0.566 | 0.961 | 0.476 | 0.522 |
| *SEE* | 0.146 | 0.479 | 0.131 | 0.478 | 0.507 | 0.451 | 0.143 | 0.495 | 0.473 |
| *F* | 126.821 | 18.711 | 157.579 | 18.879 | 14.809 | 23.494 | 131.351 | 16.338 | 19.689 |
| *R*^2^_pred_ | 0.1145 | 0.2483 | 0.6473 | 0.2838 | 0.1599 | 0.2836 | 0.752 | 0.2127 | 0.22 |
| *SEP* | 0.549 | 0.551 | 0.543 | 0.571 | 0.582 | 0.551 | 0.557 | 0.586 | 0.57 |
| Nc | 3 | 1 | 3 | 1 | 3 | 1 | 3 | 1 | 1 |
| **Field Contribution** | | | | | | | | | |
| S | 0.665 | 0.332 | 0.232 | 0.171 | 0.2 | 0.137 | 0.195 | 0.128 | 0.108 |
| E | 0.335 | 0.668 | 0.345 | 0.344 | 0.403 | 0.276 | 0.24 | 0.257 | 0.217 |
| H | - | - | 0.424 | - | - | 0.197 | 0.337 | - | 0.155 |
| D | - | - | - | 0.485 | - | 0.39 | - | 0.363 | 0.306 |
| A | - | - | - | - | 0.397 | - | 0.228 | 0.253 | 0.214 |

*R*^2^_cv_ = Cross-validated correlation coefficient after the leave-one-out procedure; *R*^2^_ncv_ = Non-cross-validated correlation coefficient; *SEE* = Standard error of estimate; *F =* Ratio of *R*^2^_ncv_ explained to unexplained = *R*^2^_ncv_/(1 − *R*^2^_ncv_); *R*^2^_pred_ = Predicted correlation coefficient for the test set of compounds; *SEP*= Standard error of prediction; Nc = Optimal number of principal components. S = steric, E = electrostatic, H = hydrophobic, D = H-bond donor, A = H-bond acceptor.

**Table S6.** Summary of QSAR results of ligand-based model for AT13387 Derivatives.

|  | **CoMFA** | **CoMSIA** | | | | | | | |
| --- | --- | --- | --- | --- | --- | --- | --- | --- | --- |
|  | **SE** | **SE** | **SEH** | **SED** | **SEA** | **SEHD** | **SEHA** | **SEDA** | **SEHDA** |
| *R*^2^_cv_ | 0.604 | 0.633 | 0.655 | 0.715 | 0.73 | 0.711 | 0.714 | 0.735 | 0.728 |
| *R*^2^_ncv_ | 0.803 | 0.828 | 0.839 | 0.892 | 0.916 | 0.892 | 0.886 | 0.959 | 0.916 |
| *SEE* | 0.411 | 0.384 | 0.371 | 0.304 | 0.275 | 0.304 | 0.313 | 0.197 | 0.268 |
| *F* | 42.783 | 50.42 | 54.817 | 86.941 | 72.459 | 86.823 | 81.574 | 111.042 | 115.04 |
| *R*^2^_pred_ | 0.7272 | 0.7848 | 0.6369 | 0.7013 | 0.4886 | 0.5979 | 0.4311 | 0.5223 | 0.5398 |
| *SEP* | 0.583 | 0.561 | 0.544 | 0.494 | 0.493 | 0.498 | 0.495 | 0.501 | 0.483 |
| Nc | 2 | 2 | 2 | 2 | 3 | 2 | 2 | 4 | 2 |
| **Field Contribution** | | | | | | | | | |
| S | 0.596 | 0.354 | 0.221 | 0.179 | 0.236 | 0.135 | 0.176 | 0.128 | 0.115 |
| E | 0.404 | 0.646 | 0.414 | 0.322 | 0.432 | 0.251 | 0.321 | 0.247 | 0.213 |
| H | - | - | 0.366 | - | - | 0.207 | 0.283 | - | 0.176 |
| D | - | - | - | 0.499 | - | 0.408 | - | 0.441 | 0.356 |
| A | - | - | - | - | 0.332 | - | 0.220 | 0.184 | 0.140 |

*R*^2^_cv_ = Cross-validated correlation coefficient after the leave-one-out procedure; *R*^2^_ncv_ = Non-cross-validated correlation coefficient; *SEE* = Standard error of estimate; *F =* Ratio of *R*^2^_ncv_ explained to unexplained = *R*^2^_ncv_/(1 − *R*^2^_ncv_); *R*^2^_pred_ = Predicted correlation coefficient for the test set of compounds; *SEP*= Standard error of prediction; Nc = Optimal number of principal components. S = steric, E = electrostatic, H = hydrophobic, D = H-bond donor, A = H-bond acceptor.

**Table S7.** Summary of QSAR Results of receptor-based model for AT13387 Derivatives.

|  | **CoMFA** | **CoMSIA** | | | | | | | | |
| --- | --- | --- | --- | --- | --- | --- | --- | --- | --- | --- |
|  | **SE** | **SE** | **SHE** | **SED** | **SEA** | **SEHD** | | **SEHA** | **SEDA** | **SEHDA** |
| *R*^2^_cv_ | 0.252 | 0.323 | 0.302 | 0.136 | 0.309 | 0.144 | | 0.267 | 0.123 | 0.132 |
| *R*^2^_ncv_ | 0.963 | 0.808 | 0.968 | 0.518 | 0.764 | 0.506 | | 0.965 | 0.473 | 0.474 |
| *SEE* | 0.188 | 0.406 | 0.175 | 0.628 | 0.45 | 0.636 | | 0.183 | 0.657 | 0.656 |
| *F* | 123.233 | 44.262 | 142.603 | 23.612 | 33.952 | 22.57 | | 129.872 | 19.769 | 19.816 |
| *R*^2^_pred_ | 0.4267 | 0.6202 | 0.1726 | 0.0961 | 0.568 | 0.129 | | 0.1935 | 0.037 | 0.0692 |
| *SEP* | 0.842 | 0.762 | 0.814 | 0.841 | 0.77 | 0.837 | | 0.834 | 0.847 | 0.843 |
| Nc | 4 | 2 | 4 | 1 | 2 | 1 | | 4 | 1 | 1 |
| **Field Contribution** | | | | | | | | | | |
| S | 0.403 | 0.348 | 0.178 | 0.197 | 0.281 | 0.147 | 0.15 | | 0.155 | 0.123 |
| E | 0.597 | 0.652 | 0.522 | 0.404 | 0.497 | 0.302 | 0.442 | | 0.319 | 0.252 |
| H | - | - | 0.301 | - | - | 0.252 | 0.268 | | - | 0.21 |
| D | - | - | - | 0.399 | - | 0.299 | - | | 0.315 | 0.249 |
| A | - | - | - | - | 0.223 | - | 0.14 | | 0.21 | 0.166 |

*R*^2^_cv_ = Cross-validated correlation coefficient after the leave-one-out procedure; *R*^2^_ncv_ = Non-cross-validated correlation coefficient; *SEE* = Standard error of estimate; *F =* Ratio of *R*^2^_ncv_ explained to unexplained = *R*^2^_ncv_/(1 − *R*^2^_ncv_); *R*^2^_pred_ = Predicted correlation coefficient for the test set of compounds; *SEP*= Standard error of prediction; Nc = Optimal number of principal components. S = steric, E = electrostatic, H = hydrophobic, D = H-bond donor, A = H-bond acceptor.

**Table S8. Summary of QSAR Results of ligand-based model for Dihydroxylphenyl amides.**

|  | **CoMFA** | **CoMSIA** | | | | | | | |
| --- | --- | --- | --- | --- | --- | --- | --- | --- | --- |
|  | **SE** | **SE** | **SEH** | **SED** | **SEA** | **SEHD** | **SEHA** | **SEDA** | **SEHDA** |
| *R*^2^_cv_ | 0.401 | 0.376 | 0.467 | 0.622 | 0.398 | 0.645 | 0.463 | 0.593 | 0.614 |
| *R*^2^_ncv_ | 0.724 | 0.57 | 0.64 | 0.821 | 0.597 | 0.858 | 0.646 | 0.827 | 0.86 |
| *SEE* | 0.668 | 0.814 | 0.744 | 0.537 | 0.787 | 0.478 | 0.738 | 0.528 | 0.476 |
| *F* | 26.192 | 27.785 | 37.3 | 45.981 | 31.164 | 60.608 | 38.294 | 47.859 | 61.262 |
| *R*^2^_pred_ | 0.6913 | 0.3662 | 0.4027 | 0.749 | 0.4201 | 0.7717 | 0.4388 | 0.7101 | 0.6996 |
| *SEP* | 0.984 | 0.98 | 0.905 | 0.781 | 0.962 | 0.757 | 0.908 | 0.81 | 0.79 |
| Nc | 2 | 1 | 1 | 2 | 1 | 2 | 1 | 2 | 2 |
| **Field Contribution** | | | | | | | | | |
| S | 0.436 | 0.369 | 0.231 | 0.241 | 0.253 | 0.153 | 0.18 | 0.178 | 0.125 |
| E | 0.564 | 0.631 | 0.395 | 0.412 | 0.432 | 0.285 | 0.307 | 0.286 | 0.226 |
| H | - | - | 0.374 | - | - | 0.29 | 0.29 | - | 0.234 |
| D | - | - | - | 0.347 | - | 0.273 | - | 0.306 | 0.256 |
| A | - | - | - | - | 0.315 | - | 0.224 | 0.23 | 0.159 |

*R*^2^_cv_ = Cross-validated correlation coefficient after the leave-one-out procedure; *R*^2^_ncv_ = Non-cross-validated correlation coefficient; *SEE* = Standard error of estimate; *F =* Ratio of *R*^2^_ncv_ explained to unexplained = *R*^2^_ncv_/(1 − *R*^2^_ncv_); *R*^2^_pred_ = Predicted correlation coefficient for the test set of compounds; *SEP*= Standard error of prediction; Nc = Optimal number of principal components. S = steric, E = electrostatic, H = hydrophobic, D = H-bond donor, A = H-bond acceptor.

**Table S9. Summary of QSAR Results of receptor-based model for Dihydroxylphenyl amides.**

|  | **CoMFA** | **CoMSIA** | | | | | | | |
| --- | --- | --- | --- | --- | --- | --- | --- | --- | --- |
|  | **SE** | **SE** | **SEH** | **SED** | **SEA** | **SEHD** | **SEHA** | **SEDA** | **SEHDA** |
| *R*^2^_cv_ | 0.342 | 0.287 | 0.457 | 0.47 | 0.198 | 0.599 | 0.417 | 0.439 | 0.545 |
| *R*^2^_ncv_ | 0.899 | 0.918 | 0.912 | 0.854 | 0.899 | 0.929 | 0.939 | 0.927 | 0.923 |
| *SEE* | 0.414 | 0.383 | 0.387 | 0.485 | 0.426 | 0.348 | 0.331 | 0.362 | 0.361 |
| *F* | 56.445 | 50.668 | 65.567 | 58.699 | 39.905 | 82.667 | 69.366 | 57.151 | 76.358 |
| *R*^2^_pred_ | 0.6702 | 0.6499 | 0.5553 | 0.5369 | 0.7227 | 0.3923 | 0.5556 | 0.4926 | 0.4844 |
| *SEP* | 1.058 | 1.131 | 0.961 | 0.925 | 1.2 | 0.826 | 1.023 | 1.003 | 0.88 |
| Nc | 3 | 4 | 3 | 2 | 4 | 3 | 4 | 4 | 3 |
| **Field Contribution** | | | | | | | | | |
| S | 0.554 | 0.389 | 0.218 | 0.242 | 0.287 | 0.146 | 0.173 | 0.187 | 0.127 |
| E | 0.446 | 0.611 | 0.373 | 0.394 | 0.423 | 0.26 | 0.294 | 0.301 | 0.206 |
| H | - | - | 0.409 | - | - | 0.328 | 0.36 | - | 0.275 |
| D | - | - | - | 0.363 | - | 0.266 | - | 0.285 | 0.241 |
| A | - | - | - | - | 0.29 | - | 0.172 | 0.228 | 0.15 |

*R*^2^_cv_ = Cross-validated correlation coefficient after the leave-one-out procedure; *R*^2^_ncv_ = Non-cross-validated correlation coefficient; *SEE* = Standard error of estimate; *F =* Ratio of *R*^2^_ncv_ explained to unexplained = *R*^2^_ncv_/(1 − *R*^2^_ncv_); *R*^2^_pred_ = Predicted correlation coefficient for the test set of compounds; *SEP*= Standard error of prediction; Nc = Optimal number of principal components. S = steric, E = electrostatic, H = hydrophobic, D = H-bond donor, A = H-bond acceptor.
